# A pilot three-month sitagliptin treatment increases serum adiponectin level in Japanese patients with type 2 diabetes mellitus- a randomized controlled trial START-J study

**DOI:** 10.1186/1475-2840-13-96

**Published:** 2014-05-24

**Authors:** Toshiyuki Hibuse, Norikazu Maeda, Ken Kishida, Takekazu Kimura, Tomoko Minami, Eriko Takeshita, Ayumu Hirata, Yasuhiko Nakagawa, Susumu Kashine, Akemi Oka, Masumi Hayashi, Hitoshi Nishizawa, Tohru Funahashi, Iichiro Shimomura

**Affiliations:** 1 Department of Internal Medicine, Suita Municipal Hospital, 2-13-20, Katayamacho, Suita, Osaka 564-0082, Japan; 2 Department of Metabolic Medicine, Graduate School of Medicine, Osaka University, 2-2 B-5, Yamada-oka, Suita, Osaka 565-0871, Japan; 3 Kishida Clinic, 5-6-3, Honmachi, Toyonaka, Osaka 560-0021, Japan; 4 Department of Clinical Trial Management, Suita Municipal Hospital, 2-13-20, Katayamacho, Suita, Osaka 564-0082, Japan; 5 Department of Metabolism and Atherosclerosis, Graduate School of Medicine, Osaka University, 2-2-B, Yamada-oka, Suita, Osaka 565-0871, Japan

**Keywords:** Sitagliptin, Adiponectin, DPP4 inhibitor, Inctrein, Diabetes, Oxidative stress

## Abstract

**Background:**

The dipeptidyl-peptidase-IV (DPP-4) inhibitors, including sitagliptin, are used for the treatment of type 2 diabetes mellitus (T2DM). Adiponectin, an adipocyte-derived circulating protein, has anti-atherosclerotic and anti-diabetic properties and is effectively elevated in bloodstream by thiazolidinediones, an insulin sensitizer. However, the effect of sitagliptin treatment on serum adiponectin level in T2DM has not fully elucidated in Japanese T2DM patients. The aim of the present study was to examine the effect of sitagliptin treatment on serum adiponectin levels in T2DM subjects.

**Methods:**

Twenty-six consecutive Japanese T2DM outpatients were recruited between April 2011 and March 2013, and randomized into the control (conventional treatment, n = 10) group and sitagliptin treatment group (n = 16). Serum adiponectin was measured by enzyme-linked immunosorbent assay.

**Results:**

Indices of glycemic control, such as hemoglobin A1c, glycated albumin, and 1.5-anhydro-D-glucitol, were significantly improved after the three-month treatment in both the control and sitagliptin groups. Serum adiponectin level was significantly increased in sitagliptin group from 6.7 ± 0.8 to 7.4 ± 1.0 μg/mL without change of body mass index (p = 0.034), while serum adiponectin level was not altered in the control group (p = 0.601).

**Conclusion:**

In Japanese T2DM patients, serum adiponectin level was elevated by three-month treatment with sitagliptin without change of body weight.

**Trial registration:**

UMIN000004721

## Background

Adiponectin is synthesized in adipocytes and secreted into the bloodstream, where it exhibits anti-diabetic and anti-atherosclerotic effects [1]. Low circulating adiponectin concentrations impact on the pathogenesis of the metabolic syndrome and atherosclerosis [1,2].

Patients with type 2 diabetes mellitus (T2DM) are treated with calorie-restricted diet and exercise therapy, if needed, plus a variety of glucose-lowering agents. Thiazolidinediones, synthetic peroxisome proliferator-activated receptor-gamma (PPAR-γ) ligands, are known to increase serum levels of adiponectin through transcriptional activation [3,4]. Incretin-based compounds, including glucagon-like peptide-1 (GLP-1) analogues and dipeptidyl peptidase-IV (DPP-4) inhibitors, have been available for the treatment of diabetes in Japan from 2009. Exendin-4, a GLP-1 receptor agonist, has shown to promote adiponectin secretion via the protein kinase-A pathway in 3 T3-L1 adipocytes [5] and to increase circulating adiponectin levels in high fat-fed rats [6]. DPP-4 inhibitors are the new class of oral glucose-lowering agents by inhibiting the inactivation of incretin hormones. Sitagliptin is the first agent approved in DPP-4 inhibitors. Among clinical trials investigating the effect of sitagliptin on glycemic control, circulating adiponectin concentration was assessed in several trials. Interestingly, Derosa G et al demonstrated that combination therapy with sitagliptin and metformin resulted in the significant increase of adiponectin at 9 months without change of body weights in the Italian multicenter, randomized, double-blind, placebo controlled trial [7], while there was a report indicating no change of adiponectin level by sitagliptin [8]. In Japanese T2DM subjects, the effect of sitagliptin on adiponectin level has not been fully determined. An observational study showed that plasma adiponectin level was increased after 12-weeks of sitagliptin treatment in Japanese T2DM subjects [9]. However, the effect of incretin-based therapy on circulating adiponectin in Japanese T2DM subjects has not been tested in a randomized controlled trial. The present randomized controlled study investigated the effect of sitagliptin treatment, the first available DPP-4 inhibitor in Japan, on serum adiponectin level in Japanese T2DM subjects, compared to conventional treatment (sulfonylurea [SU] and/or biguanide drugs).

## Methods

### Participants

The study (START-J study: Effect of Sitagliptin on Adiponectin and oxidative stress in Diabetic Patients–Japan, #UMIN000004721) subjects were consecutive 26 Japanese diabetic outpatients with poor glycemic control (hemoglobin A1c [HbA1c]; 7.6 ± 0.2%), who adhered to low-calorie diet and exercise and/or received with oral hypoglycemic agents (SU and/or biguanide drugs), and were recruited at Suita Municipal Hospital from April 2011 to March 2013. We excluded from the study patients who were treated with pioglitazone, which is known to increase serum levels of adiponectin [10], α-glucosidase inhibitors, and/or insulin injection. Patients with severe renal dysfunction under peritoneal and/or blood dialysis were also excluded [11,12]. Patients who received the moderate to high dose of SU agents (glimepiride ≥2 mg/day, glibenclamide ≥1.25 mg/day, or gliclazide ≥40 mg/day), were excluded to avoid a hypoglycemia after sitagliptin administration. The Medical Ethics Committees of Suita Municipal Hospital and Osaka University approved the protocol of the study, and a signed informed consent was obtained from each participant.

### Study protocol

**Figure 1:**
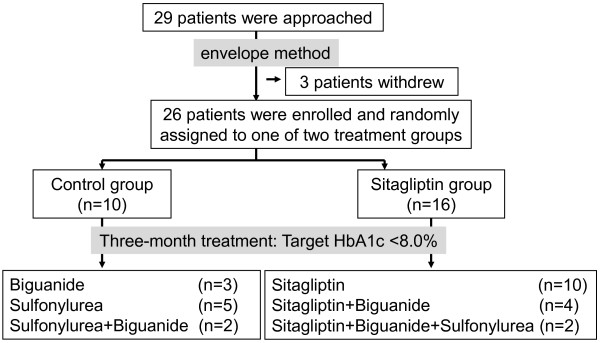
Flowchart of START-J study protocol.

This study was a single-center prospective randomized controlled trial involving T2DM outpatients. T2DM subjects were randomized into either 3-month conventional treatment (control group) or 3-month treatment with sitagliptin (dose: 25 or 50 mg/day, sitagliptin group). Various parameters were assessed at baseline and after the three-month treatment. According to the judgment of attending physician, worsening of glycemic control (HbA1c ≥8.0%) during the follow-up period was corrected by increasing the dose of test drug or adding another oral glucose-lowering agents; 1) sitagliptin group; increase the dose of sitagliptin up to 100 mg/day, or add SU agent (glimepiride up to 3 mg/day or gliclazide up to 40 mg/day) and/or biguanide (metformin up to 500 mg/day or buformin up to 100 mg/day), 2) control group; add a SU agent (glimepiride up to 3 mg/day or gliclazide up to 40 mg/day) and/or biguanide (metformin up to 500 mg/day or buformin up to 100 mg/day) (Figure 1). The attending physicians also verify glycemic control by glycoalbumin and 1.5-anhydro-D-glucitol (1.5AG). The upper limit of SU agents was kept to the recommendation of the Japan Diabetes Society. To avoid lactic acidosis and to perform clinical trial safely, the upper limit of biguanides was set up.

An oral glucose tolerance test (OGTT) was performed under overnight fasting at baseline and three-months. Under 75-g OGTT, plasma glucose and immunoreactive insulin (IRI) levels were measured at 0, 30, 60, and 120 min. The homeostasis model assessment of insulin resistance (HOMA-IR) was calculated by the formula: HOMA-IR (milliunits per liter × milligrams per deciliter) = [(fasting IRI × fasting glucose)/405]. The homeostasis model assessment of pancreatic β-cell function (HOMA-β) was calculated by the formula: HOMA-β [(milliunits per liter)/(milligrams per deciliter)] = [(fasting IRI × 360)/(fasting glucose - 63)]. The insulinogenic index represented the ratio of IRI to glucose from 0 to 30 min after glucose loading. Insulin sensitivity index was calculated by the formula: 10,000/square root of [(fasting glucose × fasting IRI) × (mean glucose × mean IRI during an OGTT)].

### Anthropometry and laboratory tests

Body height, body weight, and waist circumference (WC) were measured in standing position and body mass index (BMI) was calculated [=weight (kg)/height (m)^2^]. WC at the umbilical level was measured with a non-stretchable tape in late expiration in standing position. Systolic and diastolic blood pressures were measured with a standard mercury sphygmomanometer on the left or right arm in sitting position after taking a rest for 10 min.

Venous blood samples were collected in the morning after overnight fast to measure serum creatinine, lipids, glucose, HbA1c (Japan Diabetes Society [JDS]), glycated albumin (GA), 1.5-anhydro-D-glucitol (1.5AG), and IRI. The value of HbA1c (%) was estimated as the National Glycohemoglobin Standardization Program (NGSP) equivalent value (%), calculated by the formula HbA1c (%) = HbA1c (JDS,%) + 0.4%. Low-density lipoprotein-cholesterol (LDL-C) was calculated using the Friedewald formula [13]. For the purpose of the present study, serum samples were immediately stored at –20°C after blood collection. All frozen samples were thawed on ice and subjected to measurements for adiponectin (Adiponectin ELISA Kit, Otsuka Pharmaceutical Co., Tokushima, Japan) [10], high-sensitivity C-reactive protein (hsCRP) (N-Latex CRP II, Dade Behring Inc, Marburg, Germany), and thiobarbituric acid reactive substances (TBARS) [10] (Japan Institute for the Control of Aging, Nikken SEIL Co., Shizuoka, Japan).

### Statistical analysis

Data are presented as mean ± SEM. Differences in frequencies were examined by the *χ*^2^ test. Differences between groups were examined for statistical significance using Mann-Whitney *U* test. In all cases, p values <0.05 were considered statistically significant. All analyses were performed with the StatView software version 5.0 (HULINKS, Inc. Tokyo, Japan).

## Results

### Baseline characteristics

Among 29 patients who were informed about the aims of this clinical trial, 3 patients were withdrawn by the following reasons: one was emergently hospitalized on the disturbance of consciousness, one discontinued to attend the hospital, and one was forgotten to collect blood sample at baseline. The remaining 26 patients were randomized into two groups by the envelope method: sitagliptin (n = 16) and control (n = 10) (Figure 1). The clinical characteristics at baseline are summarized in Table 1. WC was significantly smaller in sitagliptin group than in control group. There were no significant differences in age (p = 0.16), BMI (p = 0.10), fasting glucose (p = 0.22), HbA1c (p = 0.40) and duration of diabetes (p = 0.56) between the two groups at baseline.

**Table 1:** Summary of characteristics of the study population

	**Control group (n = 10)**		**Sitagliptin group (n = 16)**	
	**Baseline**	**Three-month**	**Baseline**	**Three-month**
Age, years	56 ± 5	-	63 ± 2	-
Gender (Male/Female)	6/4	-	9/7	-
Body mass index, kg/m^2^	28.1 ± 1.4	27.4 ± 1.0	24.9 ± 1.2	24.9 ± 1.2
Waist circumference, cm	99.4 ± 2.9	97.2 ± 3.0	88.8 ± 3.4*	89.0 ± 3.2
Duration of DM, (years)	3.8 ± 1.3	-	4.8 ± 1.1	-
Fasting glucose, mg/dL	156 ± 10	141 ± 6	142 ± 6	134 ± 6
Hemoglobin A1c, (%)	7.8 ± 0.4	7.0 ± 0.2^¶^	7.5 ± 0.2	6.8 ± 0.2^¶^
Glycoalbumin, %	18.2 ± 0.8	16.2 ± 0.9^¶^	18.9 ± 0.8	16.5 ± 0.6^¶^
1.5AG, μg/mL	6.4 ± 1.2	9.7 ± 1.9^¶^	8.5 ± 1.4	12.9 ± 1.8^¶^
HOMA-IR, units	5.02 ± 2.1	3.21 ± 0.9	2.0 ± 0.3	1.7 ± 0.3
HOMA-Iβ, units	45.6 ± 12.1	43.3 ± 11.3	29.7 ± 5.7	28.4 ± 5.0
Insulinogenic index	0.11 ± 0.03	0.15 ± 0.04^¶^	0.12 ± 0.02	0.14 ± 0.04
Anti-diabetic therapy				
Diet and Excise only, n (%)	9 (90%)	0 (0%)	10 (62.5%)	0 (0%)
Sulfonylurea (SU), n (%)	0 (0%)	5 (50%)	0 (0%)	0 (0%)
Biguanide (BG), n (%)	0 (0%)	3 (30%)	4 (25%)	0 (0%)
SU and BG, n (%)	1 (10%)	2 (20%)	2 (12.5%)	0 (10%)
Sitagliptin, only, n (%)	-	-	-	10 (62.5%)
Sitagliptin and BG, n (%)	-	-	-	4 (25%)
Sitagliptin, SU and BG, n (%)	-	-	-	2 (12.5%)
Hypertension, n (%)	5 (50%)	-	13 (81%)	-
Systolic blood pressure, mmHg	129 ± 3	132 ± 5	145 ± 6	133 ± 3
Diastolic blood pressure, mmHg	78 ± 5	79 ± 3	80 ± 3	76 ± 2
Dyslipidemia, n (%)	4 (40%)	-	8 (50%)	-
Triglyceride, mg/dL	228 ± 39	185 ± 30^¶^	157 ± 26	135 ± 17
HDL-C, mg/dL	51 ± 4	50 ± 4	58 ± 2	57 ± 3
LDL-C, mg/dL	130 ± 12	124 ± 11	119 ± 7	111 ± 7
Medications				
ACEI or ARB, n (%)	2 (20%)		9 (56.3%)	
CCBs, n (%)	1 (10%)		5 (31.3%)	
Statins, n (%)	1 (10%)		4 (25%)	
Antiplatelet drugs, n (%)	0 (0%)		2 (12.5%)	

Data are mean ± SEM, n (%).

*p < 0.05 vs. control group. ^¶^p < 0.05 vs. baseline by the same treatment.

HOMA-IR, homeostasis model assessment-insulin resistance; HOMA-β, HOMA β cell function; 1.5AG, 1.5-anhydro-D-glucitol, ACEI, angiotensin-converting enzyme inhibitor; ARB, angiotensin II receptor blocker; CCB, calcium-channel blocker.

### Clinical features after three-month treatment

In sitagliptin group, all patients were started with 50 mg of sitagliptin and maintained with the same doses at the end of study. In control group, there were no patients whose medication was increased during the study. Three-month sitagliptin treatment significantly improved HbA1c, GA, and 1.5AG, compared to at baseline (Table 1, p < 0.05), without changes in BMI or WC. However, there were no significant differences in HbA1c, GA, and 1,5-AG between control and sitagliptin groups at 3 months. Significant changes were observed in insulinogenic index and serum triglyceride at 3 months in control group.

### 75 g-OGTT at baseline and after three-month treatment

**Figure 2:**
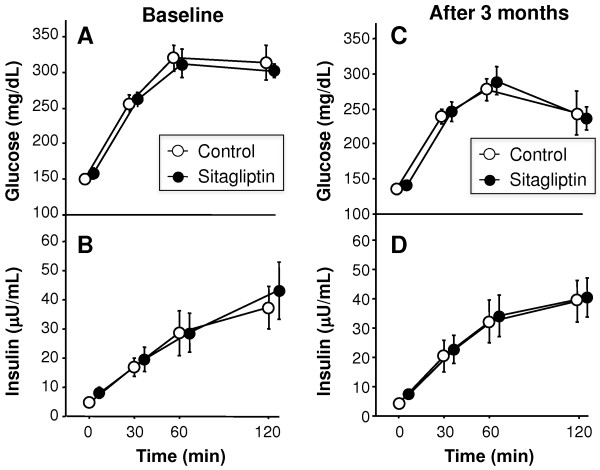
**Plasma glucose and insulin curves under 75 g-OGTT at baseline and three months.** Plasma glucose **(A)** and serum insulin **(B)** under 75-gram oral glucose tolerance test (75 g-OGTT) at baseline (**A** and **B**, respectively) and 3 months after treatment (**C** and **D**, respectively). Data are mean ± SEM of 9 patients of the control group and 12 patients of the sitagliptin group.

**Figure 3:**
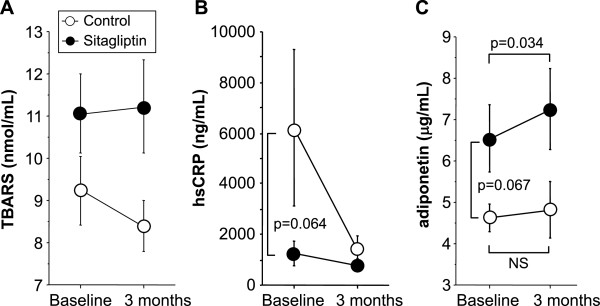
**Changes in serum TBARS, hsCRP, and adiponectin levels.** Serum levels of thiobarbituric acid-reacting substance (TBARS) **(A)**, high-sensitivity C-reactive protein (hsCRP) **(B)**, and adiponectin **(C)**. Data are mean ± SEM of 10 subjects of the control group and 16 subjects of the sitagliptin group. NS; not significant.

OGTT was performed in 21 subjects of all participants. Figure 2 shows plasma glucose and IRI levels under 75 g-OGTT before and after treatment. There were no significant changes in the glucose and insulin curves in both control (n = 9) and sitagliptin groups (n = 12) (Figure 2).Figure 3 demonstrates changes in serum levels of TBARS, hsCRP, and adiponectin. At baseline, there were no significant differences in TBARS between the control and sitagliptin groups (p = 0.121). Serum levels of hsCRP and adiponectin tended to be different in two groups, but such differences were not statistically significant (p = 0.064 and p = 0.067, respectively). Three-month treatment showed no significant changes in serum levels of TBARS and hsCRP relative to the baseline in both groups (Figure 3). Interestingly, sitagliptin treatment resulted in a significant increase of serum adiponectin level (6.7 ± 0.8 μg/mL at baseline versus 7.4 ± 1.0 μg/mL at post-treatment, p = 0.034), but there was no such change in control group (4.6 ± 0.3 μg/mL at baseline versus 4.9 ± 0.7 μg/mL at post-treatment, p = 0.601) (Figure 3).

## Discussion

The main finding of this study was the significant increase in serum adiponectin level in T2DM patients at the end of three-month sitagliptin therapy without change of body weight.

Cardiovascular disease (CVD) is the leading cause of morbidity and mortality in patients with diabetes. Lowering blood glucose level is an important aspect of management for T2DM. However, there is still debate whether anti-diabetic therapy targeting at normalization of HbA1c can actually reduce cardiovascular events, at least in patients with advanced T2DM [14,15]. The impact of the intensive glycemic control on cardiovascular complications is still under debate. The United Kingdom Prospective Diabetes Study (UKPDS) was the first clinical trial to provide a key evidence for the importance of intensive glucose-lowering therapy in individuals with newly diagnosed T2DM [16,17]. Actually, insulin- or SU-based intensive glucose-lowering treatment significantly reduced the risk of major microvascular endpoints [16]. However, such intensive treatment for T2DM did not reach a statistical significance for reduction of macrovascular CVD events compared with the conventional treatment [16]. Furthermore, the Action to Control Cardiovascular Risk in Diabetes (ACCORD) study, the Action in Diabetes and Vascular Disease (ADVANCE) study, and the Veterans Affairs Diabetes Trial (VADT) have shown no beneficial effects of the intensive glucose-lowering treatment on primary cardiovascular endpoints in T2DM [18-20]. Taken together, these clinical trials suggest that physicians need to integrate such pleiotropic effects on cardiovascular disease to improve the quality of drug-therapy decisions.

Adiponectin possesses numerous physiological properties such as anti-atherosclerotic, anti-diabetic, and anti-inflammatory effects [1,2]. Subjects with hypoadiponectinemia tend to develop into cardiovascular events and diabetes [21,22]. It is therefore believed that the intervention to increase circulating adiponectin level may provide the protection against atherosclerosis and diabetes. Activation of PPAR-γ by thiazolidinediones increases circulating adiponectin concentration at the transcriptional level [3,4]. Importantly, PPAR-γ agonist failed to reduce the atherosclerotic area in adiponectin-deficient mice under apolipoprotein E (ApoE) knockout background [23], suggesting that thiazolidinediones protect against atherosclerosis partly through its enhancing effect on adiponectin. Actually, the PROactive ("PROspective pioglitAzone Clinical Trial In macroVascular Events") study reported that pioglitazone, a PPAR-γ agonist, improved cardiovascular outcome in a high-risk population of T2DM patients [24,25].

Incretin-based therapies, GLP-1 receptor (GLP-1R) agonists and DPP-4 inhibitors, enhance the glucose-dependent insulin secretion and optimize the management of glycemic control without hypoglycemia and weight gain [26]. DPP-4 inhibitors were clinically introduced in 2006, with sitagliptin (Glactiv®, Januvia®, Xelevia™, Tesavel®) as the first agent [27], followed by vildagliptin, saxagliptin, linagliptin, teneligliptin, alogliptin, and anagliptin. Based on their efficacy (which is not inferior to sulfonylureas), low risk of hypoglycemia, body-weight neutrality, and mostly once-daily dosing, DPP-4 inhibitors seem to fulfill the aforementioned requirements [28]. The beneficial effect of DPP-4 inhibitors on cardiovascular events has not been determined, however several meta-analysis studies have recently reported the safety of DPP-4 inhibitors on cardiovascular events [29-32]. The present study, as a randomized clinical trial, demonstrated for the first time that short-term sitagliptin treatment resulted in a significant increase in serum adiponectin level in T2DM patients without changes in BMI and WC, which may be an important finding linked to the pleiotropic effects of sitagliptin in the prevention of cardiovascular events. Current result may imply that oral administration of sitagliptin is an effective and well-tolerated treatment option for cardiovascular management of T2DM patients.

Several experimental studies showed that treatment with a GLP-1 analogue [5,6] or DPP4 inhibitors [33,34] increased serum adiponectin levels, but such GLP-1 analogue-mediated increase of adiponectin has remained uncertain in human subjects [35]. Furthermore, the mechanism for the increase of adiponectin by sitagliptin has not been clarified. Increase of systemic and/or local oxidative stress reduces adiponectin production [36] and thus there was a possibility that sitagliptin increased adiponectin directly or indirectly through the decrease of oxidative stress in T2DM patients. However, no significant change of TBARS was observed in present short-term sitagliptin treatment (Figure 3A). Reduction of body weight and visceral fat also result in the elevation of serum adiponectin level [1,2]. In the present study, there were no significant changes of BMI and WC before and after sitagliptin treatment (Table 1). Effect of sitagliptin or metformin added to pioglitazone monotherapy in T2DM patients was explored in the multicenter, randomized, double-blind clinical trial in Italy [8]. This study was performed during 12 months and clinical parameters were assessed every 3 months. Plasma adiponectin level was not altered in sitagliptin group, but was significantly elevated at 12 months in metformin group. Plasma hsCRP levels were significantly decreased at 12 months in both groups. Pioglitazone has been shown as a significant enhancer of adiponectin [3,4] and thus sitagliptin-dependent increase of adiponectin may be masked by pioglitazone. Similar group also investigated the effect of the sitagliptin plus metformin combination therapy or metformin monotherapy in Italy [7]. They showed the significant elevation of adiponectin at 9 and 12 months in the combination therapy with sitagliptin and metformin and at 12 months in the metformin monotherapy. Interestingly, significant reduction of body weight was observed at 12 months in both groups, indicating the possibility that sitagliptin possesses the elevation of circulating adiponectin prior to weight reduction. Present study also showed the sitagliptin-dependent increase of adiponectin without change of BMI. Recently, the 6 months treatment with vildagliptin, one of DPP4 inhibitors, has been shown to decrease body weight, WC, and fat mass, and to increase adiponectin level [37], suggesting the possibility that long-term sitagliptin treatment may reduce visceral fat mass and elevate serum adiponectin level. Further experimental and larger scale clinical studies including long-term follow-up studies are required to determine the mechanism of the beneficial effects of sitagliptin on serum adiponectin.

The present study demonstrated that three-month sitagliptin treatment increased circulating adiponectin levels in T2DM patients without changes in BMI, although several study limitations have arisen below. The previous and present findings may provide the possibility that sitagliptin potentially possesses pleiotropic and protective effects against atherosclerotic cardiovascular events.

### Study limitations

The present study has several limitations. The study population was small and the number of sitagliptin group was larger than that of the control group, although subjects were randomized by the envelope method. Selection of medicine was left on the attending physician. WC at baseline was significantly larger in control group than in sitagliptin group. There were several imbalances in age, BMI, blood pressure, lipid profile, and medications between control and sitagliptin groups. In addition, the treatment period was limited to three-months only. Collectively, crossover clinical trials and/or long-term/large-scale studies are desired to confirm the effects of sitagliptin on serum adiponectin level and cardiovascular outcome.

## Abbreviations

DPP-4: Dipeptidyl-peptidase-IV; T2DM: Type 2 diabetes mellitus; GLP-1: Glucagon-like peptide-1; PPAR-γ: Peroxisome proliferator-activated receptor-gamma; SU: Sulfonylurea; OGTT: Oral glucose tolerance test; IRI: Immunoreactive insulin; HOMA-IR: Homeostasis model assessment of insulin resistance; WC: Waist circumference; BMI: Body mass index; GA: Glycated albumin; 1,5-AG: 1,5-anhydro-D-glucitol; hs-CRP: High-sensitivity C-reactive protein; TBARS: Thiobarbituric acid reactive substances; CVD: Cardiovascular disease.

## Competing interests

Tohru Funahashi is a member of the “Department of Metabolism and Atherosclerosis”, a sponsored course endowed by Kowa Co. Ltd. Toshiyuki Hibuse, Ken Kishida, Norikazu Maeda, and Hitoshi Nishizawa are consultants and promotional speakers for ONO PHARMACEUTICAL CO., LTD. Iichiro Shimomura serves as an adviser for ONO PHARMACEUTICAL CO., LTD, and has received lecture fees and research funds from MSD K.K. and ONO PHARMACEUTICAL CO., LTD.

## Authors’ contributions

TH researched and analyzed the data. TH, KK, and NM wrote and reviewed/edited the manuscript. TH, NM, KK, and HN participated in the concept and design of the study, and interpretation of the data. TH, SK and MN analyzed the data. TK, TM, ET, AH, YN, and SK recruited the patients and collected the data. AO, and MH assigned the patients. TF and IS contributed to the discussion. All authors read and approved the final version of the manuscript.
